# Supplementary material for: Characterization of an mRNA-Encoded Antibody Against Henipavirus
Source: Curr Issues Mol Biol. 2025 Jul 4;47(7):519. doi: 10.3390/cimb47070519 (PMC12293297; doi:10.3390/cimb47070519)
Supplement: Supplementary file 1 [file cimb-47-00519-s001.zip › cimb-3622394-supplementary.pdf]

current issues in molecular biology

MDPI

Supplementary Materials

Table S1. Sequences of the coding sequence of antibody 1E5.

Name	Sequence
1E5 H	ATGAACTTCGGGCTGTCCCTGAT-<br>TTTCCTGGTGCTGATCCTCAAGGGCGTGCAGTGCGTG-<br>CAGCTGCAGGAGAGCGGCCCTGGGGTGGTGAAGCCCTCCGAGACCCTC<br>AGCCTGACCTGCGCTGTCTCCGGGGGGTCCATTTCCGATACAT-<br>ACCGGTGGAGCTGGATCAG-<br>ACAGCCCCCCGGCAAGGGCCTGGAGTGGATCGGGTACATCTACGGGTC<br>CGCCACCAGCACATACTACAACCCCAGCCTGTCCAGCCGGGTGAC-<br>CATTAG-<br>CAAGGACATGTCCAAGAACCAGTTTAGCCTGAACCTGAACAGCGTGAC<br>CGCCGCCGACACCGCCGTGTACTACTGCGCCAGGGACTACCAG-<br>TACTACTACAGCGGCTCC-<br>TACCCCACACCCCACAACTGGTTCGACGTGTGGGGGCCTGGGGTGCTG<br>GTGACCGTGAGCTCCGCCAGCACCAAGGGGCCTAGCGTGTTCCCAC-<br>TGGCCCCAAGCAGCAA-<br>GAGCACAAGCGGGGGCACAGCCGCCCTGGGGTGCCTGGTGAAGGATT<br>ACTTCCCTGAGCCCGTCACAGTGAGCTGGAACTCCGGCGCCCTGAC-<br>CAGCGGCGTCCACACATTCCCCGCCGTGCTGCAGAGCTCCGGCCTGTA<br>CAGCCTGAGCAGCGTGGTGACAGTGCCCAGCAGCAGCCTGGG-<br>GACACAGACTTACATCTG-<br>TAACGTGAACCACAAGCCCAGCAACACAAAGGTGGATAAGAAGGTCG<br>AGCCAAAGTCCTGCGATAAGACACAT-<br>ACCTGCCCCCCCTGCCCAGCCCCCGAGCTGCTGGGCGGGCCCAGCGTG<br>TTTCTGTTCCCCCCCAAGCCCAAGGATACCCTGATGATCAGCAG-<br>GACCCCCGAGGTGACATGCGTGGTGGTGGATGTGTCCCACGAGGACCC<br>TGAGGTGAAGTTCAACTGGTACGTGGATGGCGTGGAGGTGCACAAC-<br>GCCAAGACTAAGCCAC-<br>GGGAGGAGCAGTACAACAGCACCTACAGGGTGGTCAGCGTGCTGACC<br>GTGCCTCACCAGGACTGGCTGAACGGGAAGGAGTACAAGTG-<br>CAAGGTGAG-<br>CAACAAGGCCCTGCCCGCCCCCATTGAAAAGACTATCTCCAAGGCCAA<br>GGGCCAGCCCCGCGAGCCCCAGGTGTACACCCTGCCCCCCTTCCGG-<br>GATGAGCTGACCAA-<br>GAACCAGGTGAGCCTGACCTGCCTGGTGAAGGGGTTCTACCCCTCCGA<br>CATCGCCGTGGAGTGGGAGAGCAACGGCCAGCCCGAGAACAAC-<br>TACAAGAC-<br>CACCCCCCCCGTGCTGGACAGCGACGGCAGCTTCTTCCTGTACTCCAAG<br>CTCACAGTGGATAAGAGCAGGTGGCAGCAGGGCAAC-<br>GTGTTCAGCTGCTCCGTGATGCAC-<br>GAGGCCCTGCATAACCACTACACACAGAAGTCCCTGAGCCTGAGCCCA<br>GGGAAGTGA
1E5 L	ATGGA-<br>TAGCCAGGCCCAGGTGCTGATGCTGCTGCTGCTCTGGGTGAGCGG-<br>GACATGCGGCGACATCCAGATGACCCAGTCCCCCAGCTCCCTGAGCGC<br>CAGCGTCGGCGATAGGGTGACCATCACCTGCCGGGCCAGCCAGGG-<br>GATCATCGACTAC-<br>CTGAGCTGGTACCAGCAGAAGCCCGGCAAGGCCCCCAAGCTGCTGATC<br>AGCACCGCCTCCAACCTGGAGAGCGGGGTGCCCAGCCGGTTCAGCGG-<br>GAGCGGCTCCGG-<br>CACAGAGTTCACCCTGACAATCAGCAGCCTGCAGCCCGAGGACTTCGC

	CACATACTCCTGTCTGCAGGGGTACACCACACCCTACACATTCGGG-<br>CAGGGGACAAAGGTG-<br>GAGATCAAGAGGACAGTGGCCGCCCCCAGCGTGTTCATCTTCCCCCCC<br>AGCGATGAACAGCTCAAGTCCGGCAC-<br>CGCCAGCGTGGTGTGCCTGCTGAACAACTTC-<br>TACCCACGGGAGGCCAAGGTGCAGTGGAAGGTGGATAACGCCCTGCA<br>GAGCGGCAACAGCCAGGAGAGCGTGACCGAGCAGGA-<br>TAGCAAGGACAGCAC-<br>TTACAGCCTGAGCTCCACACTGACCCTGTCCAAGGCCGACTACGAGAA<br>GCACAAGGTGTACGCCTGCGAGGTCACACACCAGGGCCTGAG-<br>CAGCCCCGTGACCAAGTCCTTCAACAGGGGGGAGTGTTGA

Table S2. Sequences of 5' UTR used in this study.

Name	Sequence
HBA1	ACTCTTCTGGTCCCCACAGACTCAGAGAGAACCCACC
HBA1(△Top)	AGGTCCCCACAGACTCAGAGAGAACCCACC
Rps27a	CTTTTCGATCCGCCATCTGCGGTGGAGCCGCCACCAAA
Rps27a(△Top)	CGATCCGCCATCTGCGGTGGAGCCGCCACCAAA
Rps25	CTTTTTGTCCGACATCTTGACGAGGCTGCGGTGTCTGCTGC-<br>TATTCTCCGAGCTTCGCA
Rps25(△Top)	GTCCGACATCTTGACGAGGCTGCGGTGTCTGCTGC-<br>TATTCTCCGAGCTTCGCA
HBM	AGAGCACGTCAGGCCGCCACC
FTH1	GCCAGACGTTCTTCGCCGAGAGTCGTCGGGGTTTCCTGCTTCAACAG-<br>TGCTTGGAC-<br>GGAACCCGGCGCTCGTTCCCCACCCCGGCCGGCCGCCCATAGCCAGCC<br>CTCCGTCACCTCTTCACCGCACCCTCG-<br>GACTGCCCCAAGGCCCCCGCCGCCGCTCCAGCGCCGCGCAGCCACCGC<br>CGCCGCCGCCGCCTCTCCTTAGTCGCCGCCACC
GADPH	GCTCTCTGCTCCTCCTGTTCGACAGTCAGCCGCATCTTCTTTT-<br>GCGTCGCCAGCCGAGCCACATCGCTCAGACACC
mRpL4	AGTGGCCTTGACCTCCCGCGGCGTGGGAGGCTGCGCGGCG
DH1	GGAATATATTAAAAGCCACC
DH2	GGGGATACCTAGCAGCCACC
DH3	GGAAATTATTTATAGCCACC
DH4	GGGACAAGATATCAGCCACC
DH5	GGAATTTATTAAAAGCCACC
DH6	GGGATAGAAACAGAACAGAAAATAGCCACC
DH7	GGGAAAGAAAAAGAACAGACAATAGCCACC
DH8	GGGAAAGAAACAGAACAGACAATAGCCACC
DH9	GGGAAAGATACAGGACAGAAAAAAGCCACC
DH10	GGGAAAGAAATAGGACAGAAAACAGCCACC
DH11	GGTATTATCTAGGAAAGAAACAGGACAGAAAAAAGCCACC
DH12	GGTATTATCTAGGAAAGAAACAGGACAAAAAAAAGCCACC
DH13	GGTATAATCTAGGAAAGAAACAGGACAGAAAAAAGCCACC
DH14	GGTACTATCTAGGAAAGAAACAGGACATAAAACAGCCACC
DH15	GGTACTATCTAGGAAATAAACAGGACAGAAAAAAGCCACC
DH16	GGGAAAGACAAGAAACATATACAAAAGAAACAAGACAGAAAA-<br>TAGCCACC

DH17	GGGAAAGAGAAGAAACACATACAAAAGAAACAAGACAGAAAA-TAGCCACC
DH18	GGGAAAGACAAGAAACACATACAAAAGAAA-TAGGACAGAAAACAGCCACC
DH19	GGGAACGATAAGAAACACATATAAAAGAAACAG-GACAGAAAACAGCCACC
DH20	GGGAAAGACAAGAAACACATAAAAAAGAAACAG-GACAGAAAACAGCCACC
DL1	GGAACTACCTAGAAGCCACC
DL2	GGGTCAACCATTTAGCCACC
DL3	GGGTCAACCTATTAGCCACC
DL4	GGGTCAACCAATTAGCCACC
DL5	GGGTCAACCTAGTAGCCACC
DL6	GGAAAAGAAAGAGTACATAAAATAGCCACC
DL7	GGAAAAGAAAGAGAACATAAAATAGCCACC
DL8	GGAAAAGAAAGAGAACAGAAAATAGCCACC
DL9	GGAAAAGAAAGAGTACATAAAAAAGCCACC
DL10	GGAAAAGAAAGAGTACAGAAAAAAGCCACC
DL11	GGAAATATATAGGAAAGAAAGAGGAAATAAAATAGCCACC
DL12	GGAAATATATAGGAAAGAAAGAGGAAAAAAAATAGCCACC
DL13	GGATTTATATAGGAAAAAAAGAGGAAATAAAAAAGCCACC
DL14	GGAAATATATAGGAAAAAAAGAGGAATAAAAAAGCCACC
DL15	GGATTTATATAGGAAAGAAAGAGAAAAAAAAAAGCCACC
DL16	GGAAACGACAAGAAATACATAAAAAAGAAACAG-GACAGAAACTAGCCACC
DL17	GGGAATTACAAGAAACATATATAAAAGAAACAGGACAGAAAA-TAGCCACC
DL18	GGGAATTTCAAGAAACAAATACAAAAGAAACAGGACAGAAAA-TAGCCACC
DL19	GGGAATTACAAGAAATAAATACAAAAGAAACAGGACAGAAAA-TAGCCACC
DL20	GGGAAAGAAAAGAAATACATATAAAAGAAACAGGACAGAAAA-TAGCCACC
Moderna	GGGAAATAAGAGAGAAAAGAAGAGTAAGAAGAAATATAA-GACCCCGGCGCCGCCACC

Table S3. Sequences of 3' UTR used in this study.

**Name**	**Sequence**
HBA1	GCTGGAGCCTCGGTGGCCATGCTTCTTGCCCCTTGGGCCTCCCCCCAGCCCCTCCTCCCCTTCCTGCACCCGTACCCCCGTGGTCTTTGAATAAAGTCTGAGTGGGCGGCA
Rps27a	CTGTATGAGTTAATAAAAGACATGAACTAACATTTATTGTTGGGTTTTATTGCAGTAAAAAGAATGGTTTTTAAGCACCAAATTGATGGTCACACCATTTCCTTTTAGTAGTGCTACTGCTATCGCTGTGTGAATGTTGCCTCTGGGGATTATGTGACCCAGTGGTTCTGTATACCTGCCAGGTGCCAACCACTTGTAAAGGTCTTGATATTTTCAATTCTTAGACTACCTATACTTTGGCAGAAGTTATATTTAATGTAAGTTGTCTAAATATAA
Rps25	ATAGGTCCAACCAGCTGTACATTTGGAAAAATAAAACTTTATTAAA
HBM	GCCCTGTGCTGCGCAGGCCTTGGTCTGTGCCTGTCAATAAACAGAGGCCCGAACCA

FTH1	GCCTCGGGCTAATTTCCCCATAGCCGTGGGGTGACTTCCCTGGTCACCA AGGCAGTGCATGCATGTTGGGGTTTCCTTTACCTTTTCTATAAGTTGTAC CAAAACATCCACTTAAGTTCTTTGATTTGTACCATTCCTTCAAATAAAG AAATTTGGTACCCAGGTGTTGTCTTTGAGGTCTTGGGATGAATCAGAAA TCTATCCAGGCTATCTTCCAGATTCCTTAAGTGCCGTTGTTCAGTTCTAA TCACACTAATCAAAAAGAAACGAGTATTTGTATTTATTAAACTCATTAG TTTGGGCAGTATACTAAGGTGTGGCTGTCTTGGATTCAGATAGAACTAA GGGTTCCCGACTCTGAATCCAGAGTCTGAGTTAAATGTTTCCAATGGTT CAGTCTAGCTTTCACAGTTTTTATGAATAAAAGGCATTAAAGGCTGAA
GADPH	GACCCCTGGACCACCAGCCCCAGCAAGAGCACAAGAGGAAGAGAGA GACCCTCACTGCTGGGGAGTCCCTGCCACACTCAGTCCCCCACCACAC TGAATCTCCCCTCCTCACAGTTGCCATGTAGACCCCTTGAAGAGGGGA GGGGCCTAGGGAGCCGCACCTTGTCATGTACCATCAATAAAGTACCCT GTGCTCAACCA
mRpL4	TGTGAAGCACCTCTTCTGAGCCAGGCCGAGCCCCTGGCCGACTTGGGA GCCTCAGGCCCACGCCCACCCTTCGAGGAAGGTGTCACCTGGACCCCT TCATTCCACGGAGGAAGCTGAGGCCACAGGGAGCGGCCATCGCCATTG GGAAGGGGCGACTCCACGGAAAGCCCAGACGGGCTTCTGCATCCATTC CCTCTTTTTGTTTTTAAAATAAATTGTATTTTTGAATCAAGGAGGATAAA
Moderna	GCTGGAGCCTCGGTGGCCTAGCTTCTTGCCCCTTGGGCCTCCCCCCAGC CCCTCCTCCCC TTCCTGCACCCGTACCCCCGTGGTCTTTGAATAAAGTC TGAGTGGGCGGCA
